# The effect of antiviral therapy on hepatitis C virus-related thrombocytopenia: a case report

**DOI:** 10.1186/1756-0500-7-59

**Published:** 2014-01-24

**Authors:** Rita Lebano, Valerio Rosato, Mario Masarone, Marco Romano, Marcello Persico

**Affiliations:** 1 Department of Internal Medicine and Hepatology, Second University of Naples, Napoli, Italy; 2 Department of Internal Medicine and Hepatology Unit, University of Salerno, Salerno, Italy; 3 Department of Gastroenterology, Second University of Naples, Napoli, Italy

**Keywords:** HCV, Autoimmune thrombocytopenic purpura, Antiviral therapy, Steroids

## Abstract

**Background:**

Autoimmune thrombocytopenic purpura is an immunological disorder characterized by increased platelet destruction due to presence of anti-platelet autoantibodies. Hepatitis C virus infection, which is one of the most common chronic viral infections worldwide, may cause secondary chronic immune thrombocytopenic purpura.

**Case presentation:**

We report a case of a 51-year-old Caucasian female with hepatitis C virus infection who developed a severe, reversible, immune thrombocytopenia. Platelet count was as low as 56.000/mm^3^, hepatitis C virus serology was positive, serum glutamic oxaloacetic transaminase, serum glutamic pyruvic transaminase and gamma-glutamyltransferase serum levels were elevated. Disorders potentially associated with autoimmune thrombocytopenic purpura were ruled out. A corticosteroid treatment was started and led to an increase in platelet count. The patient was then treated with pegylated-interferon alpha 2a and ribavirin. After four weeks of treatment hepatitis C virus - ribonucleic acid became undetectable and steroid treatment was discontinued. Six months of antiviral therapy achieved a sustained biochemical and virological response together with persistence of normal platelet count.

**Conclusion:**

In our case report hepatitis C virus seemed to play a pathogenic role in autoimmune thrombocytopenic purpura. Moreover, the successful response (negative hepatitis C virus - ribonucleic acid) to tapered steroids and antiviral therapy was useful to revert thrombocytopenia.

## Background

Autoimmune thrombocytopenic purpura (AITP) is an immunological disorder characterized by increased platelet destruction due to presence of anti-platelet autoantibodies [1]. The resulting thrombocytopenia may expose patients to life – threatening hemorrhagic complications. AITP has been classified either as a primary autoimmune disorder or as secondary to a number of underlying conditions such as lymphoproliferative disorders, myelodysplasia, autoimmune collagen vascular diseases (e.g., systemic lupus erythematosus, antiphospholipid antibody syndrome) liver disease with portal hypertension, drugs or viral infection. Thrombocytopenia has also been described in association with hepatitis C virus (HCV) which is one of the most common chronic viral infections worldwide and a major cause of cirrhosis, end-stage liver disease, and hepatocellular carcinoma [2,3]. The pathophysiology of thrombocytopenia in patients with HCV chronic infection is complex and multifactorial [4,5]. However, pathogenic mechanisms may include: hypersplenism secondary to portal hypertension when patients develop extensive fibrosis and/or cirrhosis, bone marrow suppression resulting from either HCV itself or interferon treatment, thrombopoietin (TPO) deficiency secondary to liver dysfunction and aberrations of the immune system resulting in the formation of anti-platelet antibodies and/or immunocomplexes that bind to platelets and facilitate their premature clearance. The most common target antigens for anti-platelet antibodies are glycoprotein IIb/IIIa, followed by GP IIIa, GP IIb, GP Ib then GP Ia [6]. A high prevalence of anti-HCV antibodies has recently been reported in a series of patients with AITP [7,8] and several cases of AITP have been reported in patients with chronic hepatitis C [9]. Therefore it is likely that HCV may play a role in the pathogenesis of AITP. The standard of care for HCV infection is the treatment with pegylated-interferon and ribavirin, which could be associated with a number of adverse events. The most frequently observed side effects are hematological and require dose reduction or treatment cessation. Autoimmune thrombocytopenia, thrombotic thrombocytopenic purpura and autoimmune hemolytic anemia have been observed in association with PEG-interferon use [8,10-16].

We report the case of a 51 - year – old Caucasian female who developed a severe, reversible, immune thrombocytopenia associated with hepatitis C virus infection. We described also our treatment schedule tapering simultaneously steroids and standard antiviral therapy with pegylated-interferon - alpha 2a (PEG-IFN – α 2a) and ribavirin on the basis of platelet count and hepatitis C virus - ribonucleic acid (HCV-RNA) levels.

## Case presentation

### Case report

**Figure 1:**
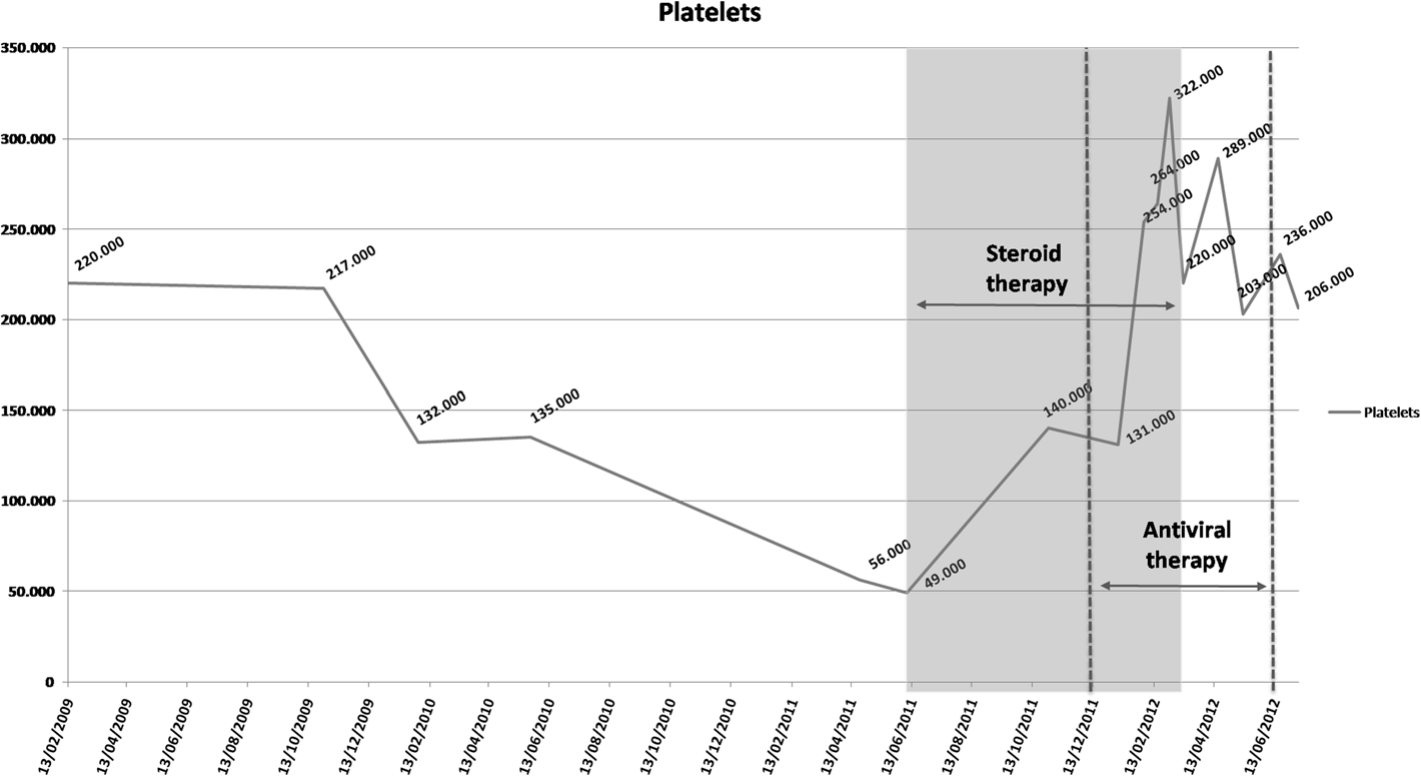
Patient’s platelet count during pre-treatment and treatment period.

A 51 - year – old Caucasian female was admitted to our Division of Hepatology because of chronic HCV infection. She showed a little increase of serum glutamic oxaloacetic transaminase (AST) and of serum glutamic pyruvic transaminase (ALT) levels, approximately two times the upper limit of the normal range, platelet count (PLT) about 132,000/mm^3^, absence of splenomegaly, anti-HCV positivity and positive serum HCV-RNA, genotype 3a. After 4 months, there was a decrease in platelet count (PLT 56.000/mm^3^) with a further increase in AST and ALT levels. At physical examination there were no ecchimosis or petechiae. Prothrombin time, albumin, pseudocholinesterase (PCHE) were all within the normal range. Abdominal ultrasonography showed a liver with normal size and moderate steatosis, portal vein of 10 mm and spleen of 11.5 cm. The use of drugs potentially causing platelet lysis was ruled out. Peripheral blood smear showed normal count of erythrocytes which were normochromicnormocytic, normal leucocytes count, decreased platelet count without platelet aggregates suggestive of a pseudo-thrombocytopenia. Concomitant lymphoproliferative disorders or autoimmune collagen vascular diseases were excluded based on negative anti-nuclear antibodies, anti mitochondrial antibodies, anti smoot-muscle antibodies, anti-liver-kidney microsome antibodies, anti thyroid autoantibodies, antiphospholipid antibodies, cryoglobulins and rheumatoid factor. Anti-platelet antibodies were found to be positive and the diagnosis of HCV associated AITP was reached. The patient started a corticosteroid therapy with prednisone 1 mg/kg/die for the first month and with dose tapering in the next three months until 5 mg/die. Platelet count increased to 140,000/mm^3^ (Figure 1). The patient continued steroid therapy with prednisone 5 mg/die. Three months later platelet count was still close to 140,000/mm^3^ and the patient started therapy with PEG-interferon-α2a and ribavirin 1200 mg/die. At the first month she achieved a rapid virological response (RVR) and the steroid therapy was discontinued. During antiviral therapy, platelet count increased to over 200,000/mm^3^ and HCV-RNA was persistently undetectable. The patient stopped the treatment after six months with a biochemical and virological response and platelet count was till over 200,000/mm^3^. After one year of follow up patients is still HCV-RNA negative and platelet count is 230,000/mm^3^.

## Discussion

A number of autoimmune diseases, such as mixed essential cryoblobulinemia, autoimmune hepatitis, systemic lupus erythematosus (LES), glomerulonephritis, rheumatoid arthritis, thyroiditis and Sjogren’s syndrome, have been reported in association with HCV infection [17,18]. Chronic HCV infection has also been associated with the development of autoimmune thrombocytopenic purpura (AITP) in absence of overt liver disease. The prevalence of autoimmune idiopathic thrombocytopenic pupura among HCV-infected subjects is controversial [8,19-25]. The overall incidence rate of AITP was higher among the HCV-infected compared with the HCV uninfected subjects. Also, the incidence rate of AITP was higher among HCV infected patients who received treatment than in HCV-infected subjects who did not. In our case, the patient developed autoimmune thrombocytopenia while she was not under treatment. After corticosteroids therapy with subsequent normalization of platelet count, she started standard antiviral therapy with PEG-interferon-α2a and ribavirin. Platelet count increased, reaching approximately 200,000/100 ml. When at the first month of antiviral therapy the HCV-RNA became negative, steroids treatment was discontinued. Six months antiviral therapy achieved a sustained biochemical and virological response together with persistence of normal platelet count, thus suggesting a causative role for HCV in AITP. In further support of a pathogenic role for HCV in AITP, a close relationship has been demonstrated between HCV viral replication and thrombocytopenia. In particular, improvements in platelet count is generally associated with a parallel decrease in HCV viral load [20,24,26] as in our case. We observed a relationship between the decrease of viral load during the treatment with PEG-interferon-α2a and ribavirin and a further increase in platelet count during antiviral treatment compared with steroid therapy alone**.** The mechanism whereby HCV may cause AITP is uncertain. HCV could determine an aberration of the immune system resulting in the formation of anti-platelet antibodies and/or immunocomplexes against platelet membrane glycoproteins that bind to platelets leading to phagocytosis of them [22,26-28]. Another pathogenic mechanism could include the specific binding between HCV and human CD81 receptor on the platelet membrane, thus causing autoantibody production against this complex [29]*.* Finally*,* Chiao [25] hypothesized that HCV may directly affect megacaryocytes thus causing their depletion. There is some difference in the clinical picture between HCV-related AITP and autoimmune idiopathic thrombocytopenia. Symptoms and sign of thrombocytopenia are less frequent in HCV positive AITP, but major bleeding is more frequent. Anticardiolipin antibodies and cryoglobulins are more frequently detected in HCV-related thrombocytopenia than in autoimmune idiopathic thrombocytopenia. In our case, the patient did not show any symptoms related to thrombocytopenia such as ecchimosis, petechiae, purpura or major bleeding, nor did she have serum cryoglobulins or autoantibodies. Therapy of AITP, independently of HCV infection, is based on intravenous immunoglobulin (IVIG) or anti-RhD Ig l [24,30,31]. While IVIG was proven effective in increasing platelet counts in 90% of cases of HCV infected patients, this did not achieve long-term response in this particular setting. Corticosteroid treatment for HCV-related AITP is controversial. Three studies [21,23,24] have shown that response to corticosteroids is significantly lower in HCV -positive than in HCV-negative patients*.* On the other hand, some case series of patients with HCV infection and chronic immune thrombocytopenic purpura have reported a greater than 50% response to treatment [22,32,33]. In our case, the patient started prednisone 1 mg/kg/day for the first month with tapering over the next three months. This led to an increase in platelet count thus making it possible to then start PEG-interferon-α2a plus ribavirin treatment. After four weeks of antiviral therapy HCV-RNA became negative, steroid treatment discontinued and the platelet count remained in the normal range during the six months of antiviral therapy and after. Splenectomy is proposed as second-line therapy. Zhang [23] and Sakuray [21] found that response to splenectomy did not differ significantly between the HCV-positive and HCV negative AITP patients. The role of antiviral therapy in HCV-related AITP in not univocal. Approximately half of adult patients with HCV-related AITP treated with interferon-α responded with a rise in platelet count [34]. Also, Iga and colleagues reported significant increase in the platelet count of 12 HCV- infected patients who were complete responders to interferon-alpha treatment, but no improvement in the platelet count of 11 patients who did not respond to interferon-alpha therapy as assessed by viral load [35]. Also in our case, after the normalization of platelet count with steroids, an antiviral treatment with PEG-interferon-α2a and ribavirin was started with a significant sustained increase in platelet count.

## Conclusion

In our case report HCV seemed to play a pathogenic role in AITP. We observed a close relationship between HCV viral replication and thrombocytopenia. In particular, improvements in platelet count is generally associated with a parallel decrease in HCV viral load. We found also a further increase in platelet count during antiviral treatment compared with steroid therapy alone. Moreover in our case the treatment schedule tapering simultaneously steroids and standard antiviral therapy together with HCV-RNA negativity was useful to revert thrombocytopenia.

## Consent

Written informed consent was obtained from the patient for publication of this case report and accompanying images. A copy of the written consent is available for review by the Editor-in-Chief of this journal.

## Abbreviations

AITP: Autoimmune thrombocytopenic purpura; HCV: Hepatitis C virus; HCV: RNA: hepatitis C virus - ribonucleic acid; AST: Serum glutamic oxaloacetic transaminase; ALT: Serum glutamic pyruvic transaminase; PLT: Platelet; PCHE: Pseudocholinesterase; LES: Systemic lupus erythematosus; PEG-IFN alpha 2a: PEG-IFN – α 2a.

## Competing interests

The authors declare that they have no competing interests.

## Authors’ contributions

MP and RL made substantial contributions reporting the clinical case and writing the manuscript; VR and MM were involved in the analysis and interpretation of the pertinent literature and in drafting the manuscript; MP and MR revised the manuscript critically for important intellectual content and gave final approval for publication. All authors read and approved the final manuscript.
